# Variations in colostrum metabolite profiles in association with sow parity

**DOI:** 10.1093/tas/txae062

**Published:** 2024-05-03

**Authors:** Julia C Vötterl, Heidi E Schwartz-Zimmermann, Frederike Lerch, Fitra Yosi, Suchitra Sharma, Markus Aigensberger, Patrick M Rennhofer, Franz Berthiller, Barbara U Metzler-Zebeli

**Affiliations:** Centre for Veterinary Systems Transformation and Sustainability, Clinical Department for Farm Animals and Food System Science, University of Veterinary Medicine, 1210 Vienna, Austria; Christian Doppler Laboratory for Innovative Gut Health Concepts of Livestock, Institute of Animal Nutrition and Functional Plant Compounds, Department for Farm Animals and Veterinary Public Health, University of Veterinary Medicine Vienna, 1210 Vienna, Austria; Christian Doppler Laboratory for Innovative Gut Health Concepts of Livestock, Institute of Animal Nutrition and Functional Plant Compounds, Department for Farm Animals and Veterinary Public Health, University of Veterinary Medicine Vienna, 1210 Vienna, Austria; Institute of Bioanalytics and Agro-Metabolomics, Department of Agrobiotechnology (IFA-Tulln), University of Natural Resources and Life Sciences, Vienna (BOKU), 3430 Tulln, Austria; Centre for Veterinary Systems Transformation and Sustainability, Clinical Department for Farm Animals and Food System Science, University of Veterinary Medicine, 1210 Vienna, Austria; Christian Doppler Laboratory for Innovative Gut Health Concepts of Livestock, Institute of Animal Nutrition and Functional Plant Compounds, Department for Farm Animals and Veterinary Public Health, >University of Veterinary Medicine Vienna, 1210 Vienna, Austria; Centre for Veterinary Systems Transformation and Sustainability, Clinical Department for Farm Animals and Food System Science, University of Veterinary Medicine, 1210 Vienna, Austria; Christian Doppler Laboratory for Innovative Gut Health Concepts of Livestock, Institute of Animal Nutrition and Functional Plant Compounds, Department for Farm Animals and Veterinary Public Health, University of Veterinary Medicine Vienna, 1210 Vienna, Austria; Department of Animal Science, Faculty of Agriculture, University of Sriwijaya, 30662 Palembang, Indonesia; Christian Doppler Laboratory for Innovative Gut Health Concepts of Livestock, Institute of Animal Nutrition and Functional Plant Compounds, Department for Farm Animals and Veterinary Public Health, University of Veterinary Medicine Vienna, 1210 Vienna, Austria; Centre for Animal Nutrition and Welfare, Clinical Department for Farm Animals and Food System Science, University of Veterinary Medicine Vienna, 1210 Vienna, Austria; Christian Doppler Laboratory for Innovative Gut Health Concepts of Livestock, Institute of Animal Nutrition and Functional Plant Compounds, Department for Farm Animals and Veterinary Public Health, University of Veterinary Medicine Vienna, 1210 Vienna, Austria; Institute of Bioanalytics and Agro-Metabolomics, Department of Agrobiotechnology (IFA-Tulln), University of Natural Resources and Life Sciences, Vienna (BOKU), 3430 Tulln, Austria; Christian Doppler Laboratory for Innovative Gut Health Concepts of Livestock, Institute of Animal Nutrition and Functional Plant Compounds, Department for Farm Animals and Veterinary Public Health, University of Veterinary Medicine Vienna, 1210 Vienna, Austria; Institute of Bioanalytics and Agro-Metabolomics, Department of Agrobiotechnology (IFA-Tulln), University of Natural Resources and Life Sciences, Vienna (BOKU), 3430 Tulln, Austria; Christian Doppler Laboratory for Innovative Gut Health Concepts of Livestock, Institute of Animal Nutrition and Functional Plant Compounds, Department for Farm Animals and Veterinary Public Health, University of Veterinary Medicine Vienna, 1210 Vienna, Austria; Institute of Bioanalytics and Agro-Metabolomics, Department of Agrobiotechnology (IFA-Tulln), University of Natural Resources and Life Sciences, Vienna (BOKU), 3430 Tulln, Austria; Centre for Veterinary Systems Transformation and Sustainability, Clinical Department for Farm Animals and Food System Science, University of Veterinary Medicine, 1210 Vienna, Austria; Christian Doppler Laboratory for Innovative Gut Health Concepts of Livestock, Institute of Animal Nutrition and Functional Plant Compounds, Department for Farm Animals and Veterinary Public Health, University of Veterinary Medicine Vienna, 1210 Vienna, Austria

**Keywords:** colostrum, fatty acids, metabolome, parity, sows

## Abstract

Information about the full spectrum of metabolites present in porcine colostrum and factors that influence metabolite abundances is still incomplete. Parity number appears to modulate the concentration of single metabolites in colostrum. This study aimed to 1) characterize the metabolome composition and 2) assess the effect of parity on metabolite profiles in porcine colostrum. Sows (*n* = 20) were divided into three parity groups: A) sows in parity 1 and 2 (*n* = 8), B) sows in parity 3 and 4 (*n* = 6), and C) sows in parity 5 and 6 (*n* = 6). Colostrum was collected within 12 h after parturition. A total of 125 metabolites were identified using targeted reversed-phase high-performance liquid chromatography-tandem mass spectrometry and anion-exchange chromatography-high resolution mass spectrometry. Gas chromatography additionally identified 19 fatty acids (FAs). Across parities, colostrum was rich in creatine and creatinine, 1,3-dioleyl-2-palmitatoylglycerol, 1,3-dipalmitoyl-2-oleoylglycerol, and sialyllactose. Alterations in colostrum concentrations were found for eight metabolites among parity groups (*P* < 0.05) but the effects were not linear. For instance, colostrum from parity group C comprised 75.4% more valine but 15.7%, 34.1%, and 47.9% less citric, pyruvic, and pyroglutamic acid, respectively, compared to group A (*P* < 0.05). By contrast, colostrum from parity group B contained 39.5% more spermidine than from group A (*P* < 0.05). Of the FAs, C18:1, C16:0, and C18:2 n6 were the main FAs across parities. Parity affected four FAs (C18:3n3, C14:1, C17:0ai, and C17:1), including 43.1% less α-linolenic acid (C18:3n3) in colostrum from parity group C compared to groups A and B (*P* < 0.05). Signature feature ranking identified 1-stearoyl-2-hydroxy-*sn*-glycero-3-phosphatidylcholine and the secondary bile acid hyodeoxycholic acid as the most discriminative metabolites, showing a higher variable importance in the projection score in colostrum from parity group A than from groups B and C. Overall, results provided a comprehensive overview about the metabolome composition of sow colostrum. The consequences of the changes in colostrum metabolites with increasing parity for the nutrient supply of the piglets should be investigated in the future. The knowledge gained in this study could be used to optimize feeding strategies for sows.

## Teaser

In this study, we characterized the metabolome composition in colostrum of sows which were in different parities. Only a small fraction of metabolites was different among parities, which may have been linked to the nutritional status and body reserves of the sows in different parities.

## INTRODUCTION

Adequate intake of colostrum by the newborn piglet is essential for its immediate survival and development (Quesnel and Farmer, 2019). Colostrum serves multiple purposes as it is the first lactocrine signal, which is critical for neonatal thermoregulation, passive immunity, and stimulation of intestinal maturation (Quesnel and Farmer, 2019; Greiner et al., 2022). Apart from the macronutrients, it contains a range of bioactive substances (e.g., immunoglobulins, growth factors, peptides, oligosaccharides, fatty acid (FA)-derived molecules, steroids, and microRNAs; Settachaimongkon et al., 2023). Factors affecting the immunological compound (i.e., immunoglobulins) in porcine colostrum have been investigated in the past (Maciag et al., 2022; Deng et al., 2023). By contrast, our knowledge about the full spectrum of metabolites present in colostrum is still incomplete. Metabolomics is a valuable tool that enables the simultaneous detection of hundreds of low-molecular-weight metabolites in biological samples, including colostrum and milk (Tan et al., 2018; Settachaimongkon et al., 2023). In using these techniques, more than 400 metabolites have been for instance listed for the colostrum of ruminants in an open data bank (https://lmdb.ca/). Recent studies analyzing the metabolome of porcine colostrum identified 123 and 116 small molecular metabolites (Tan et al., 2018; Settachaimongkon et al., 2023). Various factors, including breed, gestation diet, health status, and environment, seem to influence the colostrum composition (Picone et al., 2018; Quesnel and Farmer, 2019; Metzler-Zebeli, 2021). For instance, some evidence exists that the parity number of sows affects the composition of the FA profile and nonvolatile polar metabolites in porcine colostrum, too, but previous findings were inconsistent (Luise et al., 2018; Settachaimongkon et al., 2023). The lipid fraction in the colostrum plays a vital role in the immediate postnatal development and health of the newborn (Lauritzen et al., 2001; Metzler-Zebeli, 2021). Therefore, it is important to understand potential changes in the colostrum lipids with increasing parity of the sow. Accordingly, we hypothesized that the parity would affect metabolites related to FA synthesis in porcine colostrum, whereas metabolites related to amino acid metabolism would change less with increasing parity. Therefore, this study aimed to 1) characterize the global colostrum metabolome and 2) assess the effect of parity on colostrum metabolite profiles.

## MATERIALS AND METHODS

### Ethics Statement

All procedures involving animal handling and treatment were approved by the institutional ethics committee of the University of Veterinary Medicine Vienna and the National authority according to the Law for Animal Experiments, Tierversuchsgesetz (BMWFW-68.205/00936-V3b/2019).

### Animals, Housing, and Sampling

Twenty sows (Swiss Large White) between first and sixth parity were used in two consecutive replicate batches with 10 sows each. Sows were retrospectively divided into three parity groups based on chemometric analysis of the metabolome data: A) sows in parity 1 (gilts; *n* = 2) and 2 (*n* = 6), B) sows in parity 3 (*n* = 4) and 4 (*n* = 2), and C) sows in parity 5 (*n* = 2) and 6 (*n* = 4). Sows were balanced for parity over the replicate batches. The sows belonged to the sow herd of the pig facility from the University of Veterinary Medicine Vienna (‘VetFarm’) and were fed and handled according to the standard protocol. Sows were handled similarly in both replicate batches. Five days before farrowing, sows were moved into free-ranging farrowing pens (BeFree, Schauer, Agrotonic, Prambachkirchen, Austria; 2.3 × 2.6 m) as described in Metzler-Zebeli et al. (2023). The pens were equipped with partly slatted flooring, a feeder, and a drinker. The farrowing pen also comprised a floor-heated nest for the piglets. Straw was provided for nesting behavior and environmental enrichment. Artificial lightning was provided from 0800 to 1430 hours and the room temperature was kept between 19 and 23 °C. Sows had free access to water. During gestation, sows were fed the regular commercial gestation diet (Supplementary Table S1). During the first trimester of gestation, the feed amount was adjusted to balance the body condition of the sows. Sows received about 2.1 kg of gestation diet twice daily at 0800 and 1430 hours in the last trimester of gestation. When moved to the farrowing room, sows were fed the commercial lactation diet from 5 days before farrowing twice a day (about 1.12 kg/meal; Table 1, Supplementary Table S1). Additionally, sows received a dietary supplement (0.5 kg/day) rich in antioxidants to stimulate their appetite and flaxseeds (0.5 kg/day) that were soaked in water. The diets met or surpassed the current recommendations for nutrient requirements (Gesellschaft für Ernährungsphysiologie [GfE], 2006; NRC, 2012). Samples from each diet were taken at the start of each replicate batch and stored at −20 °C until analysis. The parturition process was supervised, and all sows farrowed in a time window of 48 h. Colostrum was collected within 1 h postpartum after ensuring that the newborn piglets could suckle. Sows were not restrained during the procedure. Therefore, colostrum was collected in the presence of the piglets. It was collected from all teats and pooled per sow; in total approximately 6 mL per sow. Only small amounts of about or less than 0.5 mL could be obtained from each teat. Colostrum samples were kept on ice after collection and stored at −20 °C. The litter performance, including litter size, piglet losses, and the body weight of piglets postpartum, was recorded.

**Table 1.** Nutrient and FA composition of lactation diet, dietary supplement, and linseeds

	Lactation diet^1^	Dietary supplement^2^	Linseeds^3^
Analyzed nutrient content, % dry matter			
Dry matter, %	89.0	92.7	95.1
Crude ash	5.5	10.7	3.4
Crude protein	17.9	16.8	22.6
Crude fiber	5.8	4.8	7.2
Neutral-detergent fiber^4^	17.3	12.0	16.7
Acid-detergent fiber	6.7	5.5	10.3
Acid-detergent lignin	1.8	1.0	4.0
Crude fat	5.2	15.9	43.8
Nitrogen-free extract	65.7	52.0	23.0
Starch	47.3	18.6	4.4
Sugar	5.5	13.5	2.5
Metabolizable energy^4^, MJ/kg	14.7	15.7	21.0
FA composition (% of total FAs)			
C6:0	ND	0.5	ND
C8:0	ND	5.3	ND
C10:0	0.1	4.1	0.1
C12:0	0.3	29.7	ND
C12:1	1.3	1.7	0.1
C14:0	0.3	11.7	0.1
C15:0	0.1	0.1	ND
C16:0	16.6	11.7	8.3
C16:1 n-7	0.2	0.5	0.1
C18:0	2.9	2.1	3.6
C18:1 *cis*/*trans*-9	22.3	11.9	18.6
C18:2 n-6t	51.1	17.9	15.6
C18:3 n-3	3.9	2.3	53.0
C20:0	0.3	0.1	0.1
C20:1 *cis*-11 n-9	0.3	0.1	0.1
Other FAs^5^	0.3	0.3	0.2
Saturated FAs	20.6	65.3	12.3
Unsaturated FAs	79.2	34.4	87.6
Monounsaturated FAs	24.1	14.2	18.9
Polyunsaturated FAs	55.0	20.2	68.7
Omega-6 FAs	51.2	17.9	15.6
Omega-3 FAs	3.9	2.3	53.0
FAs, g/100 g diet sample	3.37	12.79	31.92

^1^ZuchtsauenKorn S Vital, Garant-Tiernahrung GmbH, Poechlarn, Austria. Ingredient composition: corn, wheat, barley, soybean meal, sunflower meal, wheat bran, apple pomace, soybean oil, calcium carbonate, monocalcium phosphate, sodium chloride, magnesium phosphate, fish oil, l-cellulose, molasses. Vitamin and mineral composition per kg feed: 10,000 IU of vitamin A, 1,800 IU of vitamin D, 100 mg of Fe as iron (II) sulfate, 15 mg of Cu as copper (II) sulfate, 90 mg of Zn as zinc sulfate, 40 mg of Mn as manganese (II) oxide, 1.5 mg of I as calcium iodate, 0.4 mg of Se as sodium selenite. Technological additives: 500 FTU phytase, 2 mg of butylated hydroxyanisole, 10 mg of butylated hydroxytoluene, 2 mg of propyl gallate.

^2^Provizog Livapig, Garant-Tiernahrung GmbH, Poechlarn, Austria. Ingredients: chiccory pulp, whey fat, soybean toasted, soy protein concentrate, saccharose, barley, wheat, wheat meal, solubilized wheat, corn, soybean oil, magnesium oxide, proxymum, lactose, fish oil, rapeseed oil, sodium chloride, mono-dicalcium phosphate, calcium carbonate, curcuma, fenugreek, onion mash, grape seed juice. Vitamin and mineral composition per kg feed: 10,000 IU of vitamin A, 2,000 IU of vitamin D, 250 IU/mg of vitamin E, 160 mg of Fe as iron (II) sulfate monohydrate, 15 mg of Cu as glycine-copper chelate, 65 mg of Zn as zinc sulfate, 40 mg of Mn as manganese (II) oxide, 6.9 mg of I as potassium iodate, 0.3 mg of Se as sodium selenite. Technological additives: 706 FTU phytase, 6,600 VU xylanase, 9,000 VU endo-1,3(4)-β-gluacanase, 9,400 mg of benzoic acid, 50 mg of propyl gallate, 703 mg of sepiolite, 16,000 mg of clinoptilolite, 196 mg of natrolite-phonolite.

^3^Linseeds, Lagerhaus, Pottenstein, Austria.

^4^Values were calculated based on official guidelines from the Association of German Agricultural Analytic and Research Institute VDLUFA (Naumann and Bassler, 2012) and German Agricultural Society (Bornholdt, 2010; Spiekers et al., 2013).

^5^Other FAs represent the sum of non-classified FAs and were not used for calculating saturated and unsaturated FA content in diet samples.

### Analytical Methods

#### Proximate nutrient analysis.

Diet samples were analyzed for dry matter (VDLUFA MBII 3.1), crude ash (VDLUFA MBII 8.1), crude protein (VDLUFA MBII 4.1.2), crude fiber (VDLUFA MBII 6.1.1), neutral-detergent fiber (VDLUFA MBII 6.5.1), acid-detergent fiber (VDLUFA MBII 6.5.2), crude fat (VDLUFA MBII 5.1.1), starch (VDLUFA MBII 7.2.1), and sugar (VDLUFA MBII 7.1.1) by Futtermittellabor Rosenau (Landwirtschaftskammer Niederösterreich, Wieselburg, Austria) following official guidelines from the Association of German Agricultural Analytic and Research Institute VDLUFA (Naumann and Bassler, 2012).

#### Targeted metabolomics analyses.

Metabolites in colostrum were analyzed by six reversed-phase high-performance liquid chromatography-tandem mass spectrometric (RP-HPLC-MS/MS) methods and one anion-exchange chromatography-high resolution mass spectrometric (AIC-HR-MS) method mode as described in detail in Metzler-Zebeli et al. (2023). In short, amino acids and biogenic amines were first derivatized with phenyl isothiocyanate and then measured by RP-HPLC-MS/MS in positive ionization mode. Bile acids as well as medium and long-chain FAs were analyzed in the derivatized sample extracts by RP-HPLC-MS/MS in negative ionization mode. Lipids with reference standards were quantified in diluted derivatized sample extracts, and lipids without reference standards were semiquantitatively determined in the same colostrum extracts by RP-HPLC-MS/MS in positive ionization mode. The dilution factor of the colostrum samples was 1:15 (v:v) for nonlipids and 1:60 (v:v) for lipids. Carboxylic acids, sugar phosphates, and nucleotides were measured by AIC-HR-MS after protein precipitation with acetonitrile/water (80:20, v:v) at a sample-to-solvent ratio of 1:25 (v:v). All sample preparation and measurement methods as well as the quantification approaches are detailed in Metzler-Zebeli et al. (2023).

#### FA analysis in diet and colostrum.

The FA in diet and colostrum samples were analyzed using a one-step extraction and methylation as previously described (Khiaosa-ard et al., 2020). For preparation, 0.5 g of the dried and ground feed sample and 2 mL of internal standard heptadecanoic acid (H3500, Sigma-Aldrich, Saint Louis, MO, USA) with a concentration of 1 mg/mL dissolved in heptane (246654, Sigma-Aldrich) was treated with 3 mL of 10% methanolic hydrogen chloride. Then, the mixture was incubated in a 90 °C water bath for 2 h, cooled to room temperature and mixed with 1 mL of heptane (246654, Sigma-Aldrich). Immediately thereafter, 10 mL of 6% aqueous potassium carbonate solution was added. The mixture was thoroughly homogenized by vortex and centrifuged at 1,600 × *g* (Eppendorf Centrifuge 5810 R, Eppendorf, Hamburg, Germany) at room temperature to enable the separation of the solvent layers. The supernatant (organic solvent) was utilized for the determination of the FA composition. For the preparation of colostrum samples, 0.5 mL of sample was mixed with 5 mL internal standard consisting of glycerol trivalerate (93498, Sigma-Aldrich), methyl nonanoate (76368, Sigma-Aldrich), methyl undecanoate (94118, Sigma-Aldrich), and methyl nonadecanoate (74208, Sigma-Aldrich) dissolved in 1,4-dioxane. Thereafter, 5 mL of 5% sodium methylate solution was added to the mixture to enable transesterification of FA into FA methyl esters and thoroughly vortexed for 60 s. The reaction was stopped with 4 mL of heptane (246654, Sigma-Aldrich) and 10 mL of 15% disodium citrate solution (71635, Sigma-Aldrich). The supernatant with the organic phase was separated and utilized for the analysis on a gas chromatograph (GC-2010 Plus, Shimadzu Corp., Kyoto, Japan) equipped with a 100 m × 0.25 mm × 0.2 μm CP Sil-88 column (CP7489, Agilent Technologies, Santa Clara, CA, USA), an autosampler (AOC-20s Auto Sampler; Shimadzu Corp.), an injector (AOC-20i Auto-Injector, Shimadzu Corp.) and a flame-ionization detector (FID-2010 Plus, Shimadzu Corp.) using helium (Helium 6.0, Linde plc, Dublin, Ireland) as carrier gas (flow of 1 mL/min). The FAs were quantified by means of the internal (methyl undecanoate) and external standards following the AOAC official method (AOAC, 2012). External standards included FA methyl esters mixture (Supelco 37 Component FAME Mix, Supelco, Bellefonte, PA, USA), α-linoleic acid (LA), conjugated methyl ester (O5632, Sigma-Aldrich), and Cis/Trans FAME Mix (35079, Restek, Bellefonte). Of note, the analytical setting did not enable the differentiation of the minor 18:1 isomers and the conjugated isomers.

#### Statistical analysis.

The residuals of the targeted metabolomics and FA data were subjected to the univariate procedure (Shapiro–Wilk test) in SAS to test for normal distribution (SAS 9.4, SAS Institute Inc., Cary, NC, USA). If residuals were not normally distributed, they were converted using the Boxcox method and the Transreg procedure in SAS. Thereafter, the data were subjected to ANOVA using the MIXED procedure and a random model. The model accounted for the fixed effect of parity group and the random effect of replicate batch. The sow represented the experimental unit. Data were expressed as least squares means ± SEM and differences at *P* ≤ 0.05 and 0.05 < *P* ≤ 0.10 were defined as significant and trend, respectively. The Tukey–Kramer test was used for pairwise comparisons among least squares means. Additionally, signature features were identified using the web-based open-access platform MetaboAnalyst 5.0 (https://www.metaboanalyst.ca/). The data were first normalized by sum and log transformation. After normalization, the data were subjected to chemometrics analysis within the statistical analysis module which was based on Partial Least Squares-Discriminant Analysis (PLS-DA). The 2D score plot was used for visualization of clustering among parity groups. Hierarchical cluster (heat map and dendogram) analysis was used for visualization of metabolome and FA data across parities. Hierarchical clustering was performed based on the Ward method and using Euclidean distance metric. The top 30 metabolites were presented in the heat map. The most discriminant metabolites for each parity group were identified. The 15 discriminant metabolites were plotted according to their importance in separating the parity groups on the variable importance in the projection (VIP) scores and visualized in loading plots (only shown for component 1). The VIP score is a weighted sum of squares of the PLS-DA loadings. The scores suggest that the selected variable is significantly involved in the separation of groups (Saleem et al. 2012).

## RESULTS

### Dietary Composition and Farrowing

The composition of the lactation diet, dietary supplement, and flaxseeds is presented in Table 1. The content of ether extract was highest in flaxseeds with 43.8% DM compared to 15.9% and 5.2% DM in the dietary supplement and lactation diet, respectively. The free FA content in the lactation diet, dietary supplement, and flaxseeds amounted to 3.4, 12.8, and 31.9 g/100 g, respectively (Table 1). In the lactation diet, 20.6% of FAs were saturated FA (SFA), 24.1% were monounsaturated FA (MUFA), and 55.0% were PUFA. The dietary supplement contained 65.3%, 14.2%, and 20.2% SFA, MUFA, and PUFA, respectively. The flaxseeds comprised 12.3%, 18.9%, and 68.7% SFA, MUFA, and PUFA, respectively. Sows were clinically healthy before farrowing. Litter size and weight were similar across parity groups at farrowing (Table 2). On average, 13 piglets per litter were born alive with an average birth weight of 1.5 ± 0.26 kg.

**Table 2.** Effect of parity group on litter size and weight at birth

Parameter	Parity group^1^			SEM	*P*-value
	Group A	Group B	Group C		
Sows, n	8	6	6	—	—
Litter size	14.0	14.7	16.8	2.19	0.706
Piglets born alive	12.3	13.2	14.3	1.62	0.657
Stillborn piglets	1.1	0.3	0.7	0.51	0.542
Mummified piglets	0.1	0.3	1.2	0.39	0.169
Average birth weight, kg	1.5	1.7	1.5	0.09	0.212

Values are least squares means ± pooled SEM.

^1^Parity group A = parity 1 and 2 (*n* = 8); parity group B = parity 3 and 4 (*n* = 6); parity group C = parity 5 and 6 (*n* = 6).

### Colostrum Metabolome

The RP-HPLC-MS/MS and IC-HR-MS analyses identified 125 unique metabolites including amino acids, amines, amides and their derivatives, sugars and metabolites from carbohydrate metabolism, bile acids, carboxylic acids, purines, pyrimidines and their derivatives as well as lipids and their derivatives in the colostrum samples (Tables 3–6). Creatine, creatinine, diglycerides, triglycerides, acetyl-carnitine, sphingomyelin C16:0, sialyllactose, citric acid, and the amino acids glutamate and leucine were the predominant metabolites in colostrum. From these metabolites, the parity group affected (*P* < 0.05) the concentration of eight metabolites and tended (*P* < 0.10) to affect the concentration of two metabolites in colostrum. Differences among parity groups existed for valine, which was 75.4% higher (*P* = 0.031) in parity group C compared to groups A and B (Table 3). Moreover, colostrum from sows in parity group C contained 47.9%, 15.7%, and 34.1% less of the amino acid derivative pyroglutamic acid, citric acid (*P* < 0.05), and pyruvic acid (*P* = 0.081) compared to sows in parity group A (Tables 3 and 4). Moreover, colostrum from sows in parity group B comprised 39.5% more of the biogenic amine spermidine (Table 3; *P* = 0.040) and tended (*P* = 0.059) to contain 33.1% less glucose-1-phosphate (Table 5) compared to group A. The colostrum of sows in parity group B was richer in glyceraldehyde-3-phosphate, which was 677% higher in colostrum from parity groups A and C (*P* = 0.003; Table 5). From the lipid derivatives (Table 6), colostrum from parity group C tended (*P* = 0.058) to comprise 132% more propionyl-carnitine compared to group A, whereas colostrum from parity groups B and C comprised 49.6% more 1,2-dielaidoyl-*sn*-glycero-3-phosphatidylcholine compared to group A (*P* = 0.020). Moreover, the colostrum from parity group C was 32.8%-richer in sphingomyelin C18:1/16:0 compared to groups A and B (*P* = 0.024).

**Table 3.** Effect of parity group on concentrations of amino acids, amines, amides and derivatives in porcine colostrum.^1^

Metabolite, mg/L	Group A	Group B	Group C	SEM	*P*-value
Proteinogenic amino acids					
Alanine	3.47	2.93	1.96	0.89	0.484
Arginine	4.45	5.20	4.47	0.56	0.607
Asparagine	0.58	1.32	0.93	0.40	0.435
Cysteine	0.69	0.87	0.99	0.12	0.209
Glutamate	11.2	11.7	11.5	1.52	0.971
Glutamine	3.58	3.56	3.14	0.55	0.828
Glycine	2.60	3.63	1.52	0.71	0.177
Isoleucine	0.95	1.14	0.82	0.17	0.491
Leucine	11.1	10.7	10.5	2.50	0.982
Lysine	2.52	3.63	2.33	0.63	0.348
Methionine	0.49	0.46	0.66	0.18	0.720
Phenylalanine	2.93	2.54	1.92	0.51	0.359
Proline	4.93	4.90	4.37	0.50	0.687
Serine	3.16	3.80	3.02	0.74	0.748
Threonine	1.05	1.29	0.66	0.24	0.238
Tryptophan	0.52	0.60	0.56	0.10	0.868
Tyrosine	2.64	2.41	2.77	0.46	0.869
Valine	3.46^b^	2.96^b^	5.63^a^	0.67	0.031
Non-proteinogenic amino acids					
β-Alanine	0.35	0.30	0.49	0.17	0.758
Citrulline	0.57	0.45	0.56	0.23	0.927
Homocysteine	0.061	0.10	0.11	0.015	0.111
Ornithine	0.19	0.21	0.15	0.04	0.624
Taurine	171	152	128	28.4	0.542
Amines, amides and derivatives					
α-Aminobutyric acid	0.12	0.22	0.13	0.08	0.636
*N*-Acetyl-d-glucosamine	4.57	3.12	3.07	0.74	0.264
Asymmetric dimethylarginine	0.024	0.040	0.042	0.008	0.222
5-Aminovaleric acid	0.055	0.060	0.034	0.027	0.800
Betaine	1.73	1.70	1.40	0.57	0.894
Cadaverine	0.070	0.067	0.053	0.006	0.163
Choline	6.93	8.42	8.56	1.15	0.522
Creatine	26.2	22.8	30.1	4.48	0.552
Creatinine (g/L)	658	715	830	93.9	0.433
Dimethylamine	3.66	3.25	4.37	0.64	0.503
Ethanolamine	6.29	6.55	6.32	0.68	0.957
γ-Aminobutyric acid	0.049	0.048	0.047	0.014	0.994
*N*-2-Hydroxyethyl iminodiacetic acid	0.010	0.005	0.005	0.002	0.178
Ketoisoleucine	0.057	0.045	0.044	0.010	0.515
Methionine-alanine	8.25	7.43	9.26	1.12	0.560
Methylamine	0.31	0.34	0.30	0.07	0.939
1-Methionine-histidine	0.020	0.037	0.045	0.016	0.521
3-Methionine-histidine	0.016	0.027	0.022	0.009	0.722
Methionine sulfoxide	0.053	0.12	0.063	0.039	0.496
*t*4-Hydroxyproline	0.37	0.32	0.30	0.10	0.887
Phenylacetylglycine	0.17	0.16	0.14	0.07	0.947
Putrescine	0.10	0.13	0.12	0.02	0.555
Pyroglutamic acid	0.56^a^	0.44a^b^	0.29^c^	0.08	0.044
Pyrrolidine	0.078	0.067	0.13	0.030	0.388
Sarcosine	0.48	0.55	0.57	0.18	0.923
Spermidine	0.47^b^	0.65^a^	0.53^ab^	0.05	0.040
Spermine	1.20	1.56	1.24	0.27	0.636

Values are least squares means ± pooled SEM.

^a,b,c^Means without a common superscript differ (*P* ≤ 0.05).

^1^Parity group A = parity 1 and 2 (*n* = 8); parity group B = parity 3 and 4 (*n* = 6); parity group C = parity 5 and 6 (*n* = 6).

**Table 4.** Effect of parity group on concentrations of bile acids, carboxylic acids and other metabolites in porcine colostrum.^1^

Metabolite, mg/L	Group A	Group B	Group C	SEM	*P*-value
Bile acids					
Lithocholic acid	0.17	0.081	0.049	0.050	0.206
Hyodeoxycholic acid	0.13	0.062	0.056	0.043	0.341
Carboxylic acids					
*cis*-Aconitic acid	6.17	4.77	5.57	0.56	0.228
Benzoic acid	0.47	0.14	0.11	0.23	0.468
Butyric acid	0.31	0.081	0.18	0.079	0.151
Caproic acid	0.045	0.059	0.043	0.021	0.849
Citric acid	689^a^	597^ab^	581^b^	31.4	0.045
3,3-Dimethylbutyric acid	0.023	0.025	0.024	0.009	0.987
Fumaric acid	0.36	0.36	0.28	0.05	0.487
3-Hydroxyglutaric acid	0.47	0.48	0.37	0.04	0.134
Hippuric acid	0.93	1.07	0.64	0.25	0.537
2-Hydroxybutyric acid	0.20	0.13	0.17	0.08	0.826
3-Hydroxybutyric acid	0.21	0.16	0.37	0.11	0.417
Hydroxyglutaric acid	0.17	0.13	0.14	0.03	0.649
α-Ketoglutaric acid	1.18	1.19	1.40	0.14	0.517
Lactic acid	4.31	4.45	3.72	0.54	0.624
Malic acid	1.29	1.49	1.11	0.12	0.130
Nicotinic acid	0.022	0.023	0.022	0.006	0.985
Pyruvic acid	1.26	1.21	0.83	0.14	0.081
Stachydrine	0.14	0.13	0.15	0.03	0.956
Succinic acid	0.18	0.21	0.085	0.050	0.230
Miscellaneous					
Cortisone	0.010	0.005	0.012	0.004	0.572
Methyl phosphate	0.38	0.45	0.29	0.13	0.731

Values are least squares means ± pooled SEM.

^a,b^Means without a common superscript differ (*P* ≤ 0.05).

^1^Parity group A = parity 1 and 2 (*n* = 8); parity group B = parity 3 and 4 (*n* = 6); parity group C = parity 5 and 6 (*n* = 6).

**Table 5.** Effect of parity group on concentrations of glycans, intermediates from carbohydrate metabolism as well as purines, pyrimidines, and their derivatives in porcine colostrum.^1^

Metabolite, mg/L	Group A	Group B	Group C	SEM	*P*-value
Glycans and intermediates from carbohydrate metabolism					
Galactose	851	800	845	26.33	0.358
Galacturonic acid	13.6	19.0	16.7	1.82	0.123
Lactose	14.9	20.8	18.3	1.99	0.123
Ribose	14.1	15.0	14.3	1.54	0.919
Sialyllactose	2,281	2,565	2,015	276	0.432
d-Fructose-6-phosphate	1.82	1.41	1.42	0.54	0.810
Galactose-1-phosphate	5.62	4.00	4.82	0.82	0.371
Glucose-1-phosphate	3.44^a^	2.30^b^	2.79^ab^	0.32	0.059
Glucose-6-phosphate	2.60	3.29	3.07	0.92	0.857
Glyceraldehyde-3-phosphate	0.26^b^	1.60^a^	0.15^b^	0.24	0.003
d-Mannose-6-phosphate	1.53	1.80	1.40	0.38	0.768
Phosphoenolpyruvic acid	0.57	0.64	0.25	0.20	0.365
6-Phosphogluconic acid	14.4	13.5	10.4	1.97	0.305
d-3-Phosphoglyceric acid	4.51	4.76	3.23	0.66	0.251
Pyruvic acid	1.26^a^	1.21^ab^	0.83^b^	0.14	0.081
Ribose-5-*P*	6.45	8.38	6.17	0.99	0.298
d-Sedoheptulose-7-phosphate	3.38	3.88	3.14	0.58	0.686
Purines, pyrimidines, and derivatives					
Deoxyguanosine	0.013	0.013	0.010	0.004	0.842
Guanosine	2.39	1.68	2.34	0.39	0.399
Hypoxanthine	1.60	1.15	1.49	0.45	0.767
Inosine	5.35	4.30	5.51	1.34	0.799
Thymine	0.086	0.068	0.042	0.021	0.345
Thymidine	0.19	0.18	0.14	0.04	0.596
Uracil	9.16	6.77	10.9	4.76	0.853
Uridine	6.22	9.60	7.03	2.75	0.653
Adenosine-3,5-cyclic-monophosphate	0.13	0.15	0.17	0.046	0.858
Adenosine-5-monophosphate	3.25	2.40	2.68	0.77	0.720
Cytidine-5-monophosphate	1.15	1.00	0.96	0.16	0.659
Guanosine-3,5-cyclic-monophosphate	2.75	2.11	2.64	0.75	0.816
Guanosine-5-diphosphate	16.6	19.2	17.2	2.73	0.791
Guanosine-5-monophosphate	13.9	10.9	11.3	1.68	0.369
Inosine-5-monophosphate	1.78	1.21	0.81	0.49	0.345
Uridine-5-diphosphate	14.4	16.0	13.0	1.70	0.512
Uridine-5-monophosphate	506	520	545	77.9	0.932

Values are least squares means ± pooled SEM.

^a,b^Means without a common superscript differ (*P* ≤ 0.05).

^1^Parity group A = parity 1 and 2 (*n* = 8); parity group B = parity 3 and 4 (*n* = 6); parity group C = parity 5 and 6 (*n* = 6).

**Table 6.** Effect of parity group on concentrations of acylcarnitines, (lyso-) phosphatidylcholines, sphingomyelin, and di- and triglycerides in porcine colostrum.^1^

Metabolite, mg/L	Group A	Group B	Group C	SEM	*P*-value
Acylcarnitines					
Free carnitine	0.15	0.21	0.25	0.07	0.605
Acetyl-carnitine	26.0	26.8	39.6	4.63	0.101
Propionyl-carnitine	1.62^b^	2.17^ab^	3.76^a^	0.60	0.058
Butyryl-carnitine	2.13	2.59	3.82	0.63	0.174
Tiglyl-carnitine	0.097	0.12	0.16	0.043	0.620
Valeryl-carnitine	53.0	66.3	82.6	15.37	0.422
Glutaryl-carnitine	3.31	3.36	3.22	1.06	0.996
Hexanoyl-carnitine	0.21	0.30	0.28	0.12	0.841
Phospholipids					
1-Palmitoyl-2-hydroxy-*sn*-glycero-3-phosphatidylcholine	0.86	0.74	0.38	0.30	0.446
1-Stearoyl-2-hydroxy-*sn*-glycero-3-phosphatidylcholine	1.27	1.58	0.51	0.56	0.469
1-Oleoyl-2-hydroxy-*sn*-glycero-3-phosphatidylcholine	4.51	3.74	3.55	0.94	0.730
1,2-Dielaidoyl-*sn*-glycero-3-phosphatidylcholine	2.80^b^	4.03^a^	4.35^a^	0.38	0.020
1-Stearoyl-2-arachidonoyl-*sn*-glycero-3-phosphatidylcholine	1.64	2.58	1.94	0.56	0.473
Sphingomyelin C18:1/16:0	32.2^b^	36.0^b^	45.3^a^	3.11	0.024
Di- and triglycerides					
1,2-Dipalmitoyl-*sn*-glycerol	9.61	11.1	12.6	2.12	0.612
1,2-Dioleoyl-*sn*-glycerol	21.8	24.5	22.7	3.94	0.879
1,3-Dioleyl-2-palmitoylglycerol	3,529	2,308	3,156	508	0.263
1,3-Dipalmitoyl-2-oleoylglycerol	1,795	1,300	1,612	222	0.315

Values are least squares means ± pooled SEM.

^a,b^Means without a common superscript differ (*P* ≤ 0.05).

^1^Parity group A = parity 1 and 2 (*n* = 8); parity group B = parity 3 and 4 (*n* = 6); parity group C = parity 5 and 6 (*n* = 6).

### Colostrum FA Composition

The gas chromatography analysis identified 19 FAs in the colostrum samples (Table 7) from which only four FAs differed among parity groups. Palmitic acid (C16:0), oleic acid (C18:1 all *cis* isomer), and linoleic acid (C18:2 n6) were the predominant FAs. Parity group did not affect the total FA concentration in colostrum samples. The proportions of myristoleic acid (C14:1 *cis*-9) and the odd-numbered heptadecenoic acid (C17:1 *cis*-10) were 84.3% and 52.4% lower in colostrum of parity group B compared to groups A and C, respectively (*P* < 0.05). Moreover, colostrum from parity groups B and C contained 20.6% more of the odd-numbered C17:0ai isomer compared to parity group A (*P* = 0.040). From the PUFA, colostrum from sows in parity group C contained 43.1% less α-linolenic acid (C18:3 *n* = 3) compared to parity groups A and B (*P* = 0.045), which resulted in an overall lower proportion of omega-3 FA in colostrum from sows of parity group C compared to parity groups A and B (*P* = 0.038).

**Table 7.** Effect of parity group on proportional composition and total amount of FAs in porcine colostrum.^1^^,^^2^

Metabolite	Group A	Group B	Group C	SEM	*P*-value
FA, % of total FAs					
C12:0	0.18	0.16	0.14	0.08	0.934
C13:0	1.90	2.60	2.33	1.01	0.879
C14:0	2.63	2.75	2.52	0.26	0.837
C14:1	0.23^a^	0.032^b^	0.18^a^	0.039	0.007
C16:0	26.8	25.2	25.3	0.86	0.321
C17:0ai	1.47	1.45	1.51	0.06	0.812
C16:1	3.59	3.76	3.80	0.33	0.890
C17:0ai	0.59^a^	0.45^b^	0.43^b^	0.03	0.040
C17:1	0.69^a^	0.32^b^	0.65^a^	0.08	0.011
C18:0	5.35	5.32	5.14	0.32	0.878
C18:1 all *trans*	0.57	0.44	0.46	0.07	0.373
C18:1 all *cis*	33.7	32.6	32.7	1.645	0.852
C18:2n6	18.8	21.2	21.8	2.318	0.604
C18:3n6	0.33	0.36	0.40	0.03	0.247
C20:1n7	0.22	0.22	0.12	0.04	0.244
C18:3n3	1.30^a^	1.37^a^	0.76^b^	0.17	0.045
C20:2 (*cis* 14)	0.420	0.440	0.46	0.03	0.544
C20:3 (*cis* 8,11,14)	0.27	0.26	0.23	0.02	0.472
C20:4n6	0.88	0.60	0.83	0.12	0.247
Other FAs^3^	0.086	0.55	0.16	0.19	0.227
Saturated FAs	38.9	37.9	37.4	1.17	0.626
Unsaturated FAs	61.0	61.6	62.5	1.13	0.654
Omega-3 FAs	1.57^a^	1.63^a^	0.99^b^	0.17	0.038
Omega-6 FAs	20.0	22.1	23.0	2.27	0.606
Monounsaturated FAs	39.0	37.3	38.0	1.84	0.795
Polyunsaturated FAs	22.0	24.2	24.5	2.30	0.679
FAs, g/100g colostrum	2.11	3.45	2.36	0.86	0.522

Values are least squares means ± pooled SEM.

^a,b^Means without a common superscript differ (*P* ≤ 0.05).

^1^Parity group A = parity 1 and 2 (*n* = 8); parity group B = parity 3 and 4 (*n* = 6); parity group C = parity 5 and 6 (*n* = 6).

^2^n3 = omega-3 FA; n6 = omega-6 FA; n7 = omega-7 FA.

^3^Other FAs represent the sum of non-classified FAs and were not used for calculating saturated and unsaturated FA content in diet samples.

### Discriminative Metabolites for Parity Groups

**Figure 1.**
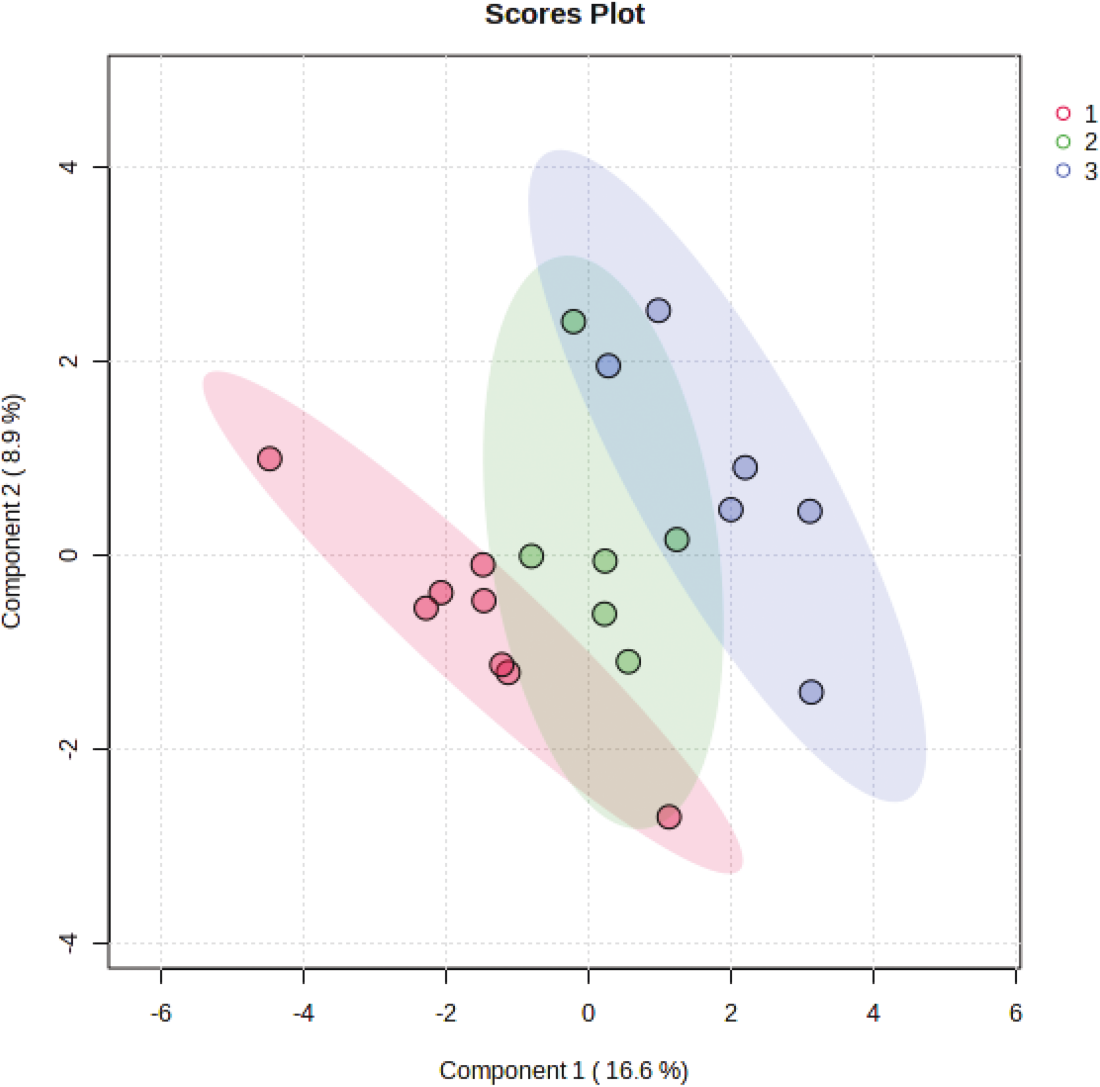
Partial least squares-discriminant analysis (PLS-DA) 2D score plot for overall comparison of metabolites in colostrum from sows in different parity numbers. Parity group A (1, parity 1 and 2, *n* = 8), B (2, parity 3 and 4, *n* = 6) and C (3, parity 5 and 6; *n* = 6).

**Figure 2.**
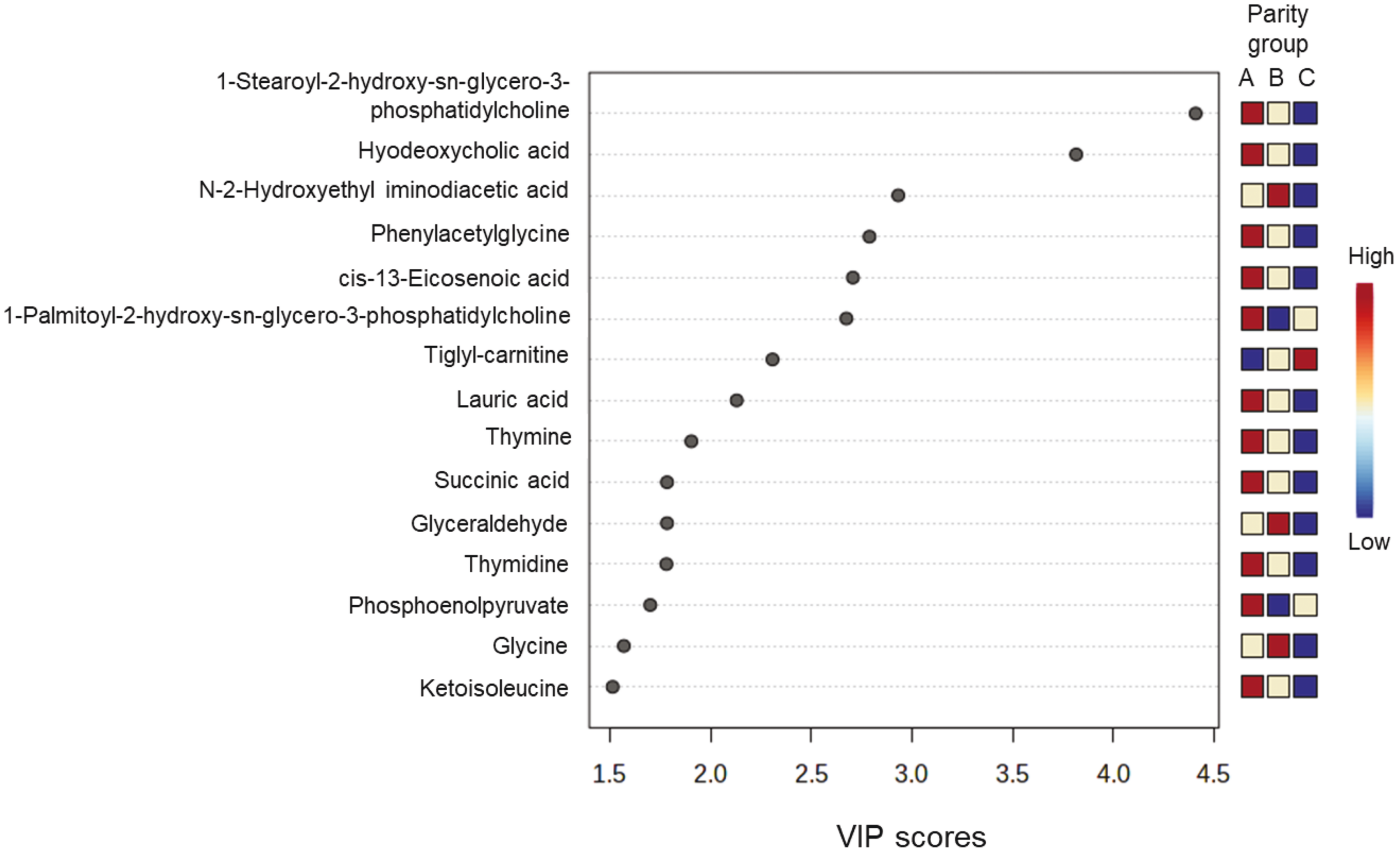
Variable importance in the projection (VIP) scores derived from the comparison of colostrum metabolite composition among parity groups A (parity 1 and 2, *n* = 8), B (parity 3 and 4, *n* = 6) and C (parity 5 and 6, *n* = 6).

**Figure 3.**
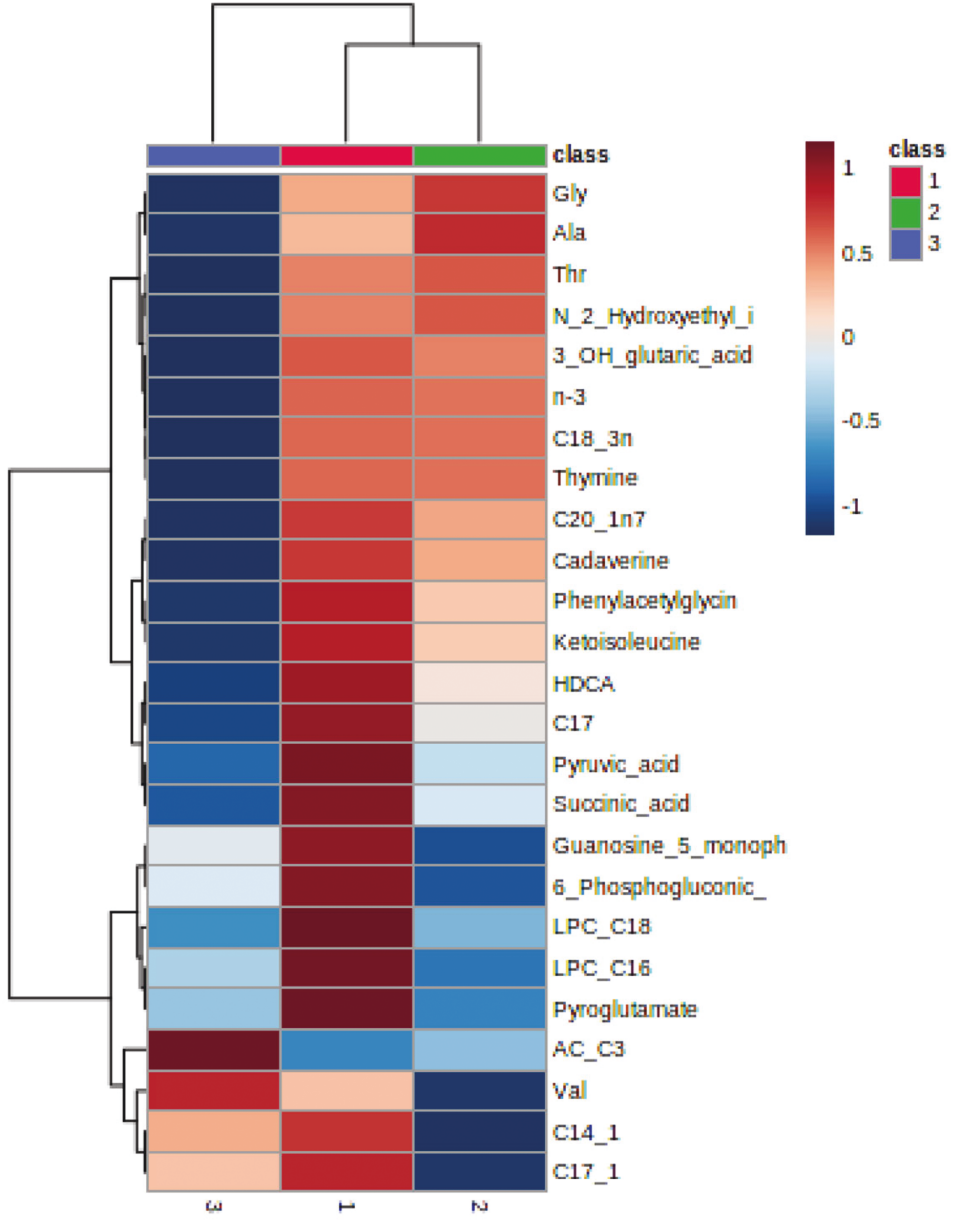
Heatmap of colostrum metabolites of hierarchical clustering results (Ward method, Euclidean distance metric) among parity groups A (class 1: parity 1 and 2, *n* = 8), B (class 2: parity 3 and 4, *n* = 6) and C (class 3: parity 5 and 6, *n* = 6). Heat map illustrated 30 most differentiating metabolites that are greater and lesser in abundance relative to the three parity groups. AC_C3, propionyl-carnitine; Ala, alanine; C14_1, C14:1; C17, C17:0; C17_1, C17:1; C18_n, C18:0; C20_1n7, C20:1n7; C20_4n6, C20:4n6; Guanosine_5_monophosph, guanosine-5ʹ-monophosphate; Gly, glycine; HDCA, hyodeoxycholic acid; 3_OH_glutaric_acid, 3-hydroxy glutaric acid; LPC_C16, lysophosphatidylcholine C16:0; LPC_C18, lysophosphatidylcholine C18:0; n-3, omega-3 fatty acids; 6_Phosphogluconic_, 6-phosphogluconic acid; N_2_Hydroxymethyl_, *n*-2-Hydroxyethyl iminodiaceticacid; TG_C16_C18_1_C16, 1,3-dipalmitoyl-2-oleoylglycerol; TG_C18_1_C16_C18_1, 1,3-dioleolyl-2-palmitoylglycerol; Thr, threonine; Val, valine.

To categorize which metabolites are most relevant for the three parity groups, we performed PLS-DA. The 2D score plots show that the metabolites in colostrum from sows of parity groups A and C cluster separately, whereas the clusters of both groups overlap with the cluster from parity group B (Fig. 1). The sources of variation for the 15 most influential metabolites were displayed based on their VIP scores (VIP > 1; Fig. 2). The VIP identified 11 metabolites (lysophosphatidylcholine C18:0, hyodeoxycholic acid, phenylacetylglycine, *cis*-13-eicosenoic acid [C20:1n7], lysophosphatidylcholine C16:0, lauric acid [C12:0], thymine, succinic acid, thymidine, phosphoenolpyruvate, and ketoisoleucine) that were discriminative for colostrum from sows of parity group A. For parity group B, concentrations of *N*-(2-hydroxyethyl)-iminodiacetic acid, glyceraldehyde, and glycine were most discriminative in colostrum, whereas for colostrum from parity group C tiglyl-carnitine was the most discriminative metabolite. The heat map with associated dendrogram of hierarchical cluster analysis visualizes the differences in concentrations across parity groups for 25 high-abundant metabolites (Fig. 3).

## DISCUSSION

In this study, we used targeted metabolomics that focused on pathways related to sugar/energy, bile acid and amino acid/nitrogen metabolism, and certain lipid fractions to characterize metabolite classes in porcine colostrum. For the interpretation of the data, it is important to note that metabolites belonging to other physiological pathways were underrepresented. Moreover, it is important to keep in mind that colostrum samples were collected after checking that the newborn piglets were capable to suckle. The number of identified metabolites is in accordance with previous reports for metabolites in porcine colostrum (Luise et al., 2018; Tan et al., 2018; Settachaimongkon et al., 2023). They originate partly from transfer from the blood and partly from mammary gland metabolism. In the first day of lactation, the blood–milk barrier is partly open which allows a greater transfer of metabolites from the blood to the first milk (Wall et al., 2015), which may be worth to investigate more closely in the future in relation to the needs of the neonate. Parity only affected a small number of metabolites and FA. The PLS-DA showed a certain clustering among parity groups, specifically between the first two parities and parities 5 and 6. Several reasons may explain the latter findings. The clustering of the parity groups may have been caused by changes in maternal and mammary gland metabolism with increasing parity but also by the actual nutritional and physiological status of the sow; data which should be collected in future research. Moreover, in future studies, it may be advisable to collect colostrum samples in a shorter time frame than we did in the present study.

Colostrum is rich in sugars, sugar phosphates, lipids, and amino acids to provide the piglet with easily accessible energy sources and building blocks (Metzler-Zebeli, 2021), which our data confirmed. Numerous metabolites detected in colostrum play roles in cell growth and differentiation as well as gut closure and protection against oxidative stress (e.g., purines and pyrimidines and biogenic amines; Schlimme et al., 2000; Gill et al., 2011; Chai et al., 2022). The detected amino acids probably originated to a great extent from the immunoglobulins in colostrum (Quesnel and Farmer, 2019). High concentrations of creatine and creatinine, and sialyllactose have been reported before for porcine colostrum (Trevisi et al., 2020; Settachaimongkon et al., 2023). Colostrum is the first feed of piglets, these concentrations may be related to piglet’s immediate needs for development, energy provision, and gut microbial colonization or are reflective for the maternal metabolism. Creatine is an important neonatal energy source (Kennaugh et al., 1997) and its association with immunoglobulin G levels has been suggested as a useful indicator for colostrum quality (Luise et al., 2020). By contrast, creatinine is a byproduct from muscle metabolism and may reflect the capacity of the sow to mobilize muscle protein for immunoglobulin production before parturition (Settachaimongkon et al., 2023). Sialyllactose is a major oligosaccharide in porcine colostrum (Trevisi et al., 2020); important to orchestrate the early microbial colonization of the gut, for instance with bifidobacteria (Wiese et al., 2018). Two triglycerides (i.e., 1,3-dioleolyl-2-palmitate and 1,3-dipalmitoyl-2-oleate) were dominant in the colostrum samples across parities, which are unique lipids found in colostrum and milk fat of mammals and are important energy sources for the neonate (Ghide and Yan, 2021). From the detected amino acids, glutamate and leucine were the most abundant amino acids. Glutamate plays a ubiquitous role in amino acid metabolism and serves as an energy source for epithelial cells (Hou and Wu, 2018). Leucine is the regulatory amino acid that stimulates muscle protein synthesis (Murgas Torrazza et al. 2010; Suryawan et al. 2012). Several metabolites from the intermediary metabolism, like pyruvic acid and citric, fumaric, succinic, and α-ketoglutaric acid (Krebs cycle) or citrulline and ornithine (urea cycle), were also present in colostrum.

Metabolites, such as butyrate, odd-numbered FA (e.g., C17:0 and C17:1), and secondary bile acids, probably originated from gut microbial metabolism. They may play a role in neonatal immune programming and survival as they are important modulators of immune functions and involved in the regulation of glucose homeostasis, lipid metabolism, and energy expenditure (Fiorucci et al., 2009; Zhang et al., 2018; Grüner and Mattner, 2021). The presence of various acylcarnitines in colostrum as carriers of long-chain FAs (Li et al., 2019) may be linked to the milk globule production of the mammary gland. Only few phospholipids were detected in the present study. They play crucial roles in the formation and stability of lipid droplets in colostrum and milk (Ding et al., 2023). The high concentration of the sphingomyelin C18:1/16:0 compared to the other phosphatidylcholines may indicate that this sphingomyelin was a major membrane component of the lipid droplets in colostrum (Fontecha et al., 2020; Da Silva et al., 2021; Venkat et al., 2022). Compared to mature milk, colostrum contains higher levels of FA with ≥18 carbons (Settachaimongkon et al., 2023), which the present data confirmed. These long-chain FAs usually originate from the diet or mobilization from maternal adipose tissue (Suarez-Trujillo et al., 2021). Accordingly, the diet and supplements fed to the sows prior to parturition in the present study were rich in oleic (C18:1) and linoleic acid (C18:2). Due to its high concentration, our results may support the importance of palmitic acid (C16:0) as an easily absorbable FA to meet the high neonatal energy needs (Luise et al., 2018; Settachaimongkon et al., 2023). Part of the palmitic acid (C16:0) probably originated from de novo synthesis in the mammary gland.

Although only a small number of metabolites differed or tended to differ among parity groups, the separate clusters in the score plot from the PLS-DA indicated significant influences of sow parity group on metabolite profiles. Of note, PLS-DA changes with parity were not linear with increasing parity number in the present study. Signature feature ranking (PLS-DA) identified discriminating metabolites in colostrum for each parity group, including, for instance, 1-stearoyl-2-hydroxy-*sn*-glycero-3-phosphatidylcholine and hyodeoxycholic acid for parity group A and tiglyl-carnitine for group C. The ANOVA did not confirm different concentrations of the discriminant metabolites among parity groups; with the exception of glyceraldehyde-3-phosphate which was indicative for parity group B. Therefore, the discriminative nature of the identified metabolites should be interpreted with care. Nevertheless, it may be worth to monitor these metabolites in future studies about parity effects on colostrum composition. In previous research (Settachaimongkon et al., 2023), parity group effects were linear and partly affected other metabolites than in the present study. For instance, creatine, glutamate, and palmitic acid (C16:0) decreased with increasing parity number in previous reports (Luise et al., 2018; Settachaimongkon et al., 2023) but not in our study. Probable reasons for the parity effects in the previous and the present study are differences in maternal and mammary gland metabolism and different degrees of mobilization and re-filling of body reserves during the gestation period with increasing parity number. Differences in the sow management, e.g., the feeding during gestation and lactation, between the current and previous studies contributed to the inter-study differences. Moreover, parity groupings and breed/linages of sows varied in the present and previous studies (Luise et al., 2018; Trevisi et al., 2020; Settachaimongkon et al., 2023). In contrast to our grouping, Settachaimongkon et al. (2023) compared primiparous sows, sows in parities 2 to 6, and sows in parity 7, whereas Luise et al. (2018) assessed differences in colostrum FA between parities 2 and 4. In the present study, parity influenced metabolite concentrations related to antioxidant capacities and cell differentiation (pyroglutamic acid and spermidine), sugar and lipid metabolism (glucose-1-phosphate, citric acid, propionyl-carnitine, glycerol-3-aldehyde, 1,2-dielaidoyl-*sn*-glycero-3-phosphatidylcholine, sphingomyelin C16:0, and the four FAs) as well as valine as proteinogenic amino acid. The elevated level of glucose-1-phosphate in colostrum from parity group A compared to group B may indicate a greater glycogenolysis in the younger sows. Moreover, the higher level of pyruvic and citric acid may suggest a better energy status of the mammary gland of these sows compared to sows in parity group C (Settachaimongkon et al., 2023), but needs further research. By contrast, the higher level of glyceraldehyde-3-phosphate, as an intermediate in glycolysis, gluconeogenesis, and pentose-phosphate-pathway (Mazurek et al., 1999), points toward a better energy metabolism in the mammary gland of sows from parity group B compared to the younger and older sows. The mammary gland of sows in parity group C, in turn, may have an increased capacity to produce fat globules (Zhang et al., 2018) as indicated by the higher concentrations of metabolites related to the lipid metabolism (e.g., propionyl-carnitine, 1,2-dielaidoyl-*sn*-glycero-3-phosphatidylcholine, and sphingomyelin C16:0). The PLS-DA identified tiglyl-carnitine as discriminative for colostrum from parity group C, which would support the assumption of a better FA activation for fat globule production by the older sows. However, triglyceride levels and total FA measured in colostrum were similar among parity groups, not supporting an improved fat globule synthesis. Pyroglutamic acid is produced during the catabolism of glutathione (Kumar and Bachhawat 2012). As it was lower in colostrum from parity group C compared to parity group A, it may hint at differences in the oxidative stress level of the mammary gland in the older sows. The higher concentration of spermidine in colostrum from parity group B was probably advantageous for neonatal survival (Chai et al., 2023).

α-Linolenic acid (C18:3 n-3) originated either from the diet or maternal fat stores. It can be speculated whether sows in parity groups A and B mobilized more body fat compared to sows in parity group C. The concentration of myristoleic acid (C14:1) was lower in colostrum from parity group B in the present study, which may indicate that the mammary tissue of the younger (group A) and older sows (group C) had a higher capacity for de novo synthesis of this FA compared to sows from parity group B. In dairy cows, the concentration of myristoleic acid has been associated with a negative energy balance (Ducháček et al., 2012; Xu et al., 2020). However, this relationship still needs to be shown for sows. Odd-chained FAs, such as heptadecanoic acid (C17:0) and heptadecenoic acid (C17:1), originate from gut microbial activity (Zhang et al., 2018). As they were lower in colostrum from parity group B (C17:0 also in parity group C), gut microbial metabolism of lipids and/or transfer to the mammary tissue (Zhang et al., 2018) may have been different between parity groups.

## CONCLUSIONS

The present metabolomics data provided a detailed overview about the complex composition of colostrum. The present data also showed that only a small fraction of metabolites was different among the three parity groups in the present study, indicating that colostrum production is favorized by the maternal metabolism around parturition. However, differences existed, suggesting for instance a better energy status and glucose utilization of the mammary gland in the younger sows. Further research should clarify the role of the time point of colostrum collection on metabolite concentrations as well as the role of the nutritional and physiological status of the sow for parity effects. The knowledge gained may be useful for the feeding management of primiparous and multiparous sows. The consequences of these changes on the supply of the metabolites to the piglets should be investigated in future studies.

## Supplementary Material

txae062_suppl_Supplementary_Table
