# Supplementary material for: Variations in colostrum metabolite profiles in association with sow parity
Source: Transl Anim Sci. 2024 May 3;8:txae062. doi: 10.1093/tas/txae062 (PMC11165641; doi:10.1093/tas/txae062)
Supplement: txae062_suppl_Supplementary_Table [file txae062_suppl_supplementary_table.docx]

Running head: Sow parity and colostrum metabolome

**Variations in colostrum metabolite profiles in association with sow parity**

Julia C. Vötterl^*,†^, Heidi E. Schwartz-Zimmermann^†,‡^, Frederike Lerch^*,†^, Fitra Yosi^*,†,^ ^§^, Suchitra Sharma^†,#^, Markus Aigensberger^†,‡^, Patrick M. Rennhofer^†,‡^, Franz Berthiller^†,‡^, Barbara U. Metzler-Zebeli^*,†,1^

^*^Unit Nutritional Physiology, Department of Biomedical Sciences, University of Veterinary Medicine, Veterinaerplatz 1, 1210 Vienna, Austria

^†^Christian Doppler Laboratory for Innovative Gut Health Concepts of Livestock, Institute of Animal Nutrition and Functional Plant Compounds, Department for Farm Animals and Veterinary Public Health, University of Veterinary Medicine Vienna, Veterinaerplatz 1, 1210 Vienna, Austria

^‡^Institute of Bioanalytics and Agro-Metabolomics, Department of Agrobiotechnology (IFA-Tulln), University of Natural Resources and Life Sciences, Vienna (BOKU), Konrad-Lorenz-Straße 20, 3430 Tulln, Austria

^§^Department of Animal Science, Faculty of Agriculture, University of Sriwijaya, 30662 Palembang, Indonesia

^#^Institute of Animal Nutrition and Functional Plant Compounds, Department for Farm Animals and Veterinary Public Health, University of Veterinary Medicine Vienna, 1210 Vienna, Austria

^1^Corresponding author: barbara.metzler@vetmeduni.ac.at

**Supplemental Table 1.** Ingredient composition of gestation and lactation diets.

| Item | Gestation diet (%)^1^ | Lactation diet (%)^2^ |
| --- | --- | --- |
| Ingredients |  |  |
| Corn | 29.99 | 35.06 |
| Barley | 28.17 | 15.00 |
| Wheat bran | 13.50 | 3.00 |
| Wheat | 10.50 | 21.50 |
| Soybean meal HP 47% | - | 9.17 |
| Sunflower meal 36% XP | 7.50 | 7.00 |
| Sugar beet pulp | 5.00 | 4.00 |
| Molasses | 1.50 | - |
| Soy oil | - | 1.20 |
| Spelt bran | 1.17 | - |
| Calcium carbonate | 0.90 | 0.70 |
| Vitamin-mineral premix | 0.60 | 0.60 |
| Vitamin-mineral premix lactation | - | 0.50 |
| Salt | 0.43 | 0.47 |
| Lysin-HCl 98 | 0.22 | 0.45 |
| Monocalciumphosphate | 0.20 | 0.72 |
| Benzoic acid | - | 0.25 |
| Phytase | 0.17 | 0.17 |
| Fish oil | 0.10 | 0.10 |
| L-Threonine | 0.05 | 0.12 |
|  |  |  |
| Calculated chemical composition (% DM) | |  |
| Dry matter (%) | 87.94 | 88.52 |
| Crude protein | 12.28 | 15.06 |
| Ether extract | 2.88 | 3.98 |
| Crude fiber | 6.18 | 4.70 |
| Crude ash | 5.16 | 5.32 |
| Starch | 42.25 | 43.33 |
| Sugar beet pulp | 3.55 | 3.02 |
| Calcium | 0.65 | 0.75 |
| Phosphorus | 0.51 | 0.55 |
| Digestible phosphorus | 0.29 | 0.39 |
| Metabolizable energy (MJ/kg) | 11.99 | 13.00 |

^1^ZuchtsauenKorn T, Garant-Tiernahrung GmbH, Poechlarn, Austria. Vitamin and mineral composition per kg feed: 10,000 IU of vitamin A, 1,800 IU of vitamin D, 120 mg of vitamin E, 100 mg of Fe as iron (II) sulfate, 15 mg of Cu as copper (II) sulfate, 90 mg of Zn as zinc sulfate, 40 mg of Mn as manganese (II) oxide, 1.5 mg of I as calcium iodate, 0.45 mg of Se as sodium selenite. Technological additives: 833 FTU phytase.

^2^ZuchtsauenKorn S Vital, Garant-Tiernahrung GmbH, Poechlarn, Austria. Vitamin and mineral composition per kg feed: 10,000 IU of vitamin A, 1,800 IU of vitamin D, 165 mg of vitamin E, 100 mg of Fe as iron (II) sulfate, 15 mg of Cu as copper (II) sulfate, 90 mg of Zn as zinc sulfate, 40 mg of Mn as manganese (II) oxide, 1.5 mg of I as calcium iodate, 0.45 mg of Se as sodium selenite. Technological additives: 833 FTU phytase.
